# Acquired Cutis Laxa

**DOI:** 10.4274/tjh.galenos.2020.2020.0121

**Published:** 2020-08-28

**Authors:** Ankur Jain

**Affiliations:** 1 Vardhman Mahavir Medical College and Safdarjung Hospital, Department of Hematology, New Delhi, India

**Keywords:** Cutis laxa, Monoclonal gammopathy of renal significance, Chemotherapy

## To the Editor,

The term monoclonal gammopathy of renal significance (MGRS) refers to paraprotein-mediated renal injury associated with an underlying clonal lymphoproliferative disorder, not meeting the criteria for multiple myeloma, Waldenstrom macroglobulinemia, or systemic lymphoma requiring therapy. Clone size (B-cell, plasma cell, or lymphoplasmacytic cell) and the resultant paraprotein are frequently small, and renal impairment results from unique physicochemical properties of the paraprotein rather than the tumor burden per se [1]. Heavy chain deposition disease (HCDD) is one of the rarest MGRS entities. Since its first description in 1993, fewer than 100 cases have been described so far [2,3,4]. HCDD results from tissue deposition of the truncated monoclonal non-amyloidogenic heavy chain (HC) with deleted constant region (CH1), most commonly IgG (γ-HCDD, IgG1>IgG3) [1,2]. Deletion of the constant region (CH1) facilitates the extracellular secretion and tissue tropism of the HC, culminating in complement-mediated end organ damage [2]. Kidneys are the most commonly affected organs, followed by skin, cardiopulmonary, gastrointestinal, vesico-urinary, and musculoskeletal systems [4]. Renal involvement frequently manifests with nephrotic-range proteinuria, hematuria, hypertension, progressive renal insufficiency, and hypocomplementemia [2,3]. Acquired cutis laxa (ACL) refers to loosening of skin due to elastotic degeneration of the dermis. Although generally associated with inflammatory conditions, a few cases of ACL have been described in association with γ-HCDD. γ-HCDD-associated ACL results from monoclonal IgG-HC dermal deposition and complement-mediated elastin degeneration [4,5].

**Figure 1:**
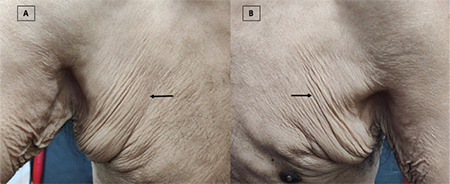
Clinical photograph of the patient showing loosening of folds of bilateral axillae (A and B). Skin biopsy findings were consistent with the diagnosis of acquired cutis laxa.

A 34-year-old Indian male developed loosening of the skin of the face, neck, and bilateral axillary folds in 2016 (Figure 1). Skin biopsy revealed loss of elastic fibers in the upper dermis, consistent with cutis laxa (CL). The patient complained of frothy urine and bilateral leg edema in 2019. A nephrology review suggested hypertension, renal insufficiency (estimated glomerular filtration rate: 15 mL/min/1.73 m^2^), inactive urine sediment, and nephrotic-range proteinuria. Kidney biopsy revealed glomerular enlargement, lobular mesangial matrix expansion with nodularity, and glomerular basement membrane splitting (silver methenamine stain). Immunohistochemistry showed linear IgG deposition along the glomerular basement membrane (immunonegative for kappa and lambda), which appeared ‘powdery’ dense on electron microscopy, confirming the diagnosis of γ-HCDD. Bone marrow aspirate showed 5% clonal plasma cells. Serum immunofixation identified an IgG-lambda monoclonal spike, and the serum free light chain ratio (sFLCr) was 0.1 (reference range: 0.26-1.65). Serum complement levels were normal and cryoglobulins were negative. The patient became dialysis-dependent and succumbed to progressive renal failure after 2 cycles of cyclophosphamide, bortezomib, and dexamethasone chemotherapy.

Due to small-sized paraprotein, about one-third of cases of γ-HCDD do not have a measurable monoclonal spike on conventional serum electrophoresis. Since clonal cells secrete monoclonal light chains in addition to the pathogenic HC, the sFLCr is frequently abnormal and could help in the diagnosis and response evaluation of γ-HCDD [2]. The renal prognosis of γ-HCDD is poor. However, clone-directed therapy, and particularly bortezomib-based therapy (with/without autologous stem cell transplantation) for associated plasma cell dyscrasia, was shown to improve renal outcomes [1,2,3]. ACL is a rare dermatological manifestation of γ-HCDD. Its presentation can precede the renal involvement by many years [5]. The current report highlights the need to meticulously search for monoclonal protein and its implicated clone in patients presenting with features of ACL. Early recognition and timely initiation of clone-directed therapy may improve renal outcomes.
